# Infectious Causes of Encephalitis and Meningoencephalitis in Thailand, 2003–2005

**DOI:** 10.3201/eid2102.140291

**Published:** 2015-02

**Authors:** Sonja J. Olsen, Angela P. Campbell, Krongkaew Supawat, Sahas Liamsuwan, Tawee Chotpitayasunondh, Somsak Laptikulthum, Akravudh Viriyavejakul, Tasanee Tantirittisak, Supoch Tunlayadechanont, Anannit Visudtibhan, Punnee Vasiknanonte, Supachai Janjindamai, Pairoj Boonluksiri, Kiatsak Rajborirug, Veerachai Watanaveeradej, Nino Khetsuriani, Scott F. Dowell, Thailand Encephalitis Surveillance Team

**Affiliations:** Thailand Ministry of Public Health–US CDC Collaboration, Nonthaburi, Thailand (S.J. Olsen, S.F. Dowell);; Centers for Disease Control and Prevention, Atlanta, Georgia, USA (S.J. Olsen, A.P. Campbell, N. Khetsuriani, S.F. Dowell);; Thailand Ministry of Health, Nonthaburi (K. Supawat);; Queen Sirikit National Institute of Child Health, Bangkok (S. Liamsuwan, T. Chotpitayasunondh);; Rajvithi Hospital, Bangkok (S. Laptikulthum);; Prasat Neurological Institute of Thailand, Bangkok (A. Viriyavejakul, T. Tantirittisak);; Ramathibodi Hospital, Bangkok (S. Tunlayadechanont, A. Visudtibhan);; Prince Songkhla University Hospital, Hat Yai, Thailand (P. Vasiknanonte, S. Janjindamai);; Hat Yai Hospital, Hat Yai (P. Boonluksiri, K. Rajborirug);; Phramongkutklao Hospital, Bangkok (V. Watanaveeradej)

**Keywords:** encephalitis, etiology, brain diseases, tropical medicine, Thailand, viruses, bacteria

## Abstract

Acute encephalitis is a severe neurologic syndrome. Determining etiology from among ≈100 possible agents is difficult. To identify infectious etiologies of encephalitis in Thailand, we conducted surveillance in 7 hospitals during July 2003–August 2005 and selected patients with acute onset of brain dysfunction with fever or hypothermia and with abnormalities seen on neuroimages or electroencephalograms or with cerebrospinal fluid pleocytosis. Blood and cerebrospinal fluid were tested for >30 pathogens. Among 149 case-patients, median age was 12 (range 0–83) years, 84 (56%) were male, and 15 (10%) died. Etiology was confirmed or probable for 54 (36%) and possible or unknown for 95 (64%). Among confirmed or probable etiologies, the leading pathogens were Japanese encephalitis virus, enteroviruses, and *Orientia tsutsugamushi*. No samples were positive for chikungunya, Nipah, or West Nile viruses; *Bartonella henselae*; or malaria parasites. Although a broad range of infectious agents was identified, the etiology of most cases remains unknown.

## Abstract 2

Although many causes were identified, most remain unknown.

Acute encephalitis is a severe neurologic syndrome that is often associated with substantial illness and death. It can be caused by any of ≈100 infectious agents that vary by geographic region. Among leading known causes of encephalitis in the United States and worldwide are arthropodborne viruses (arboviruses) and herpesviruses (*1*–*9*). Data on encephalitis in Southeast Asia are limited, but previous studies have identified Japanese encephalitis virus (JEV) and herpesviruses as common causes (*10*–*12*). Dengue virus has also been associated with encephalopathy and other neurologic findings (*4*,*13*–*15*). Over the past decade, several viral encephalitides have emerged in Southeast Asia, including Nipah virus and enterovirus 71, and led to unexpected outbreaks of neurologic disease (*4*,*16*–*23*).

Determining the etiology of encephalitis is difficult. The definition varies, and distinguishing the neurologic syndrome of encephalitis from meningoencephalitis or even meningitis or encephalopathy can be challenging (*24*,*25*). In recent studies, the etiology for most cases was not determined despite extensive testing (*1*,*6*–*12*,*26*). One characteristic of past studies is that laboratory diagnosis for encephalitis was often not complete because of lack of available diagnostics, limited scope of pathogens studied, or difficulty obtaining adequate specimens. Detection capabilities are limited by testing for only a limited core battery of pathogens or the most likely pathogens on the basis of exposure history and clinical information and by use of conventional diagnostics.

To determine the spectrum of encephalitis etiologic agents in Thailand, we conducted a prospective study and used an expanded testing approach. We used a case definition consistent with definitions used in prior studies, requiring acute brain dysfunction and evidence of inflammation and including patients who also had meningeal inflammation with an encephalitic component (meningoencephalitis) (*1*,*10*,*12*). We sought to identify etiologic pathogens for patients with a clinical syndrome consistent with encephalitis and meningoencephalitis and to describe the clinical features and outcomes associated with different causes.

## Methods

### Study Sites

Patients were recruited from 7 hospitals in Thailand: 5 in Bangkok (Queen Sirikit National Institute of Child Health, Rajvithi Hospital, Prasat Neurologic Institute, Ramathibodi Hospital, and Phramongkutkao Hospital) and 2 in the southern city of Hat Yai (Hat Yai Hospital, Prince Songkhla University Hospital). The main study physicians in each hospital were specialists in neurology or infectious diseases. During July 2003–August 2005, physicians identified potential study participants who met eligibility criteria and referred them to a study nurse, who obtained written informed consent. The protocol was approved by an institutional review board at the Centers for Disease Control and Prevention (CDC) and the Thailand Ministry of Public Health.

### Enrollment Criteria and Case Definition

Enrollment criteria for patients of any age included all of the following: 1) acute brain dysfunction requiring hospitalization (new encephalopathy [i.e., altered mental status with or without lethargy], new onset of diffuse or focal central neurologic findings, or new onset of seizures); 2) acute onset of brain dysfunction within 14 days before or 7 days after admission to a study hospital; 3) documented fever (>38°C), history of fever, or hypothermia (<35°C); and 4) clinical indication for lumbar puncture as determined by the patient’s physician. After enrollment, participants were required to meet at least 1 of the following 3 criteria to meet the case definition of acute encephalitis or meningoencephalitis: 1) abnormal findings consistent with encephalitis seen on neuroimages obtained by computed tomography (CT) scan, magnetic resonance imaging (MRI), or cranial ultrasonography; 2) abnormal findings on electroencephalogram (EEG) consistent with encephalitis; or 3) cerebrospinal fluid (CSF) pleocytosis (>15 leukocytes/mm^3^ for infants <6 weeks of age and >5 leukocytes/mm^3^ for patients >6 weeks of age). Patients found to have an alternative confirmed diagnosis before discharge that explained their signs and symptoms (e.g., metabolic encephalopathy) were excluded from further study.

### Epidemiologic Data and Specimen Collection

For each patient, study physicians completed standardized admission and discharge surveys documenting medical history, signs and symptoms, and neuroimaging and EEG results. Research nurses completed an extensive questionnaire about demographics, medical and exposure history, and laboratory results. Study nurses completed a follow-up questionnaire during each patient’s convalescent-phase visit 3–6 weeks after enrollment. The following specimens were collected: CSF (up to 6.5 mL); acute- and convalescent-phase blood (12.5–22.5 mL from children <5 years of age and 25.5 mL from all others); and oropharyngeal swab, saliva (0.7 mL), urine (10 mL), and fecal (10–20 g) specimens.

### Specimen Handling, Storage, and Testing

Specimens were tested for the presence of >30 pathogens (Table 1). After collection, specimens were immediately separated into portions for clinical testing at hospitals and research testing at reference laboratories (Technical Appendix). Within 24 hours of collection, specimens destined for reference laboratories were aliquoted for distribution to >20 laboratories at the Thailand National Institute of Health and CDC. Any specimens that could not be aliquoted within 24 hours were stored at −20°C and then at −70°C after aliquoting. Specimens from Hat Yai were transported on dry ice to Bangkok every 2–4 weeks. If CSF or serum sample volume was limited, the order of specimen testing was prioritized (Technical Appendix).

**Table 1:** Classification strategy for etiology of encephalitis, Thailand, 2003–2005*

Agent	Established cause of encephalitis	Diagnostic category		
		Confirmed	Probable	Possible
Bacteria				
* Bartonella henselae*	Yes		≥4-fold rise serum Ab titer (IFA)	
* Haemophilus influenzae*	Rare	CSF or blood culture positive CSF latex agglutination positive		
* Mycobacterium tuberculosis*	Rare	CSF culture positive		
* Mycoplasma pneumoniae*	Yes	CSF PCR positive	Serologic conversion (neg to pos) on serum Ab titer (EIA)	OP PCR positive
* Neisseria meningitidis*	Rare	CSF or blood culture positive CSF latex agglutination positive		
*Rickettsia* spp. (e.g., *Rickettsia conorii*, *Orientia tsutsugamushi*)	Yes		>4-fold rise serum Ab titer	
* Streptococcus pneumoniae*	Rare	CSF or blood culture positive; CSF latex agglutination positive		
* Treponema pallidum*	Yes	CSF VDRL positive	Blood specific treponemal Ab positive	
Other	Rare (organism dependent)	CSF or blood culture positive; CSF 16S RNA PCR positive + sequence (organism dependent)	CSF 16S RNA PCR positive + sequence (organism dependent)	Blood culture positive (organism dependent)
Fungi				
*Cryptococcus* spp.	Yes (in immunocompromised patients)	CSF culture positive; India ink positive; cryptococcal antigen positive		
Parasites				
Malaria parasites	Yes	Thick or thin smear positive in presence of malarial disease		
Other parasites (e.g., *Toxoplasma gondii*)	Yes (parasite dependent)	Detection of parasites in CSF or brain		
Viruses				
Adenoviruses	Yes (except for enteric HAdV 40, 41)	CSF PCR positive (other than HAdV 40, 41)		≥4-fold rise serum Ab titer (EIA); OP PCR positive; CSF PCR positive for HAdV 40, 41
Chikungunya virus	Rare	IgM in CSF with CHIKV PRNT titer >1:10 in CSF or serum	>4-fold rise in neutralizing Ab titers in serum	
Cytomegalovirus (Human herpesvirus 5)	Yes (mostly in immunocompromised patients)		CSF PCR positive	
Dengue virus	Rare	DENV RNA in serum or CSF; DENV IgM in serum with a DENV PRNT_90_ titer >1:20 and a DENV PRNT_90_ to JEV PRNT_90_ titer ratio >1:4		
Enteroviruses	Yes	CSF isolation in culture; CSF PCR positive		4-fold rise Ab titer
Epstein-Barr virus (human herpesvirus 4)	Yes, dependent on clinical situation		CSF PCR positive (predictive value in immunocompetent hosts is unclear)	
Herpes simplex viruses 1 and 2	Yes	CSF PCR positive		CSF IgM present
Human herpesvirus 6	Yes	CSF PCR positive		
Human herpesvirus 7	Yes (in immunocompromised patients), not well established			CSF PCR positive
HIV	Yes			Serology with Western blot positive
Influenza viruses	Rare		>4-fold rise serum Ab titer	OP PCR positive
Japanese encephalitis virus	Yes	JEV IgM in CSF; JEV IgM in serum with a JEV PRNT_90_ titer >1:20 and a JEV PRNT_90_ to DENV PRNT_90_ titer ratio >1:4	JEV IgM in serum with a JEV PRNT_90_ titer >1:20 but a JEV PRNT_90_ to DENV PRNT_90_ titer ratio <1:4	
Measles virus	Yes	CSF Ab positive		>4-fold rise serum Ab titer if no recent vaccination
Mumps virus	Yes	CSF Ab positive		>4-fold rise serum Ab titer if no recent vaccination
Nipah virus	Yes	CSF PCR positive; 4-fold rise serum Ab titer		
PIV 1, 2, and 3	No (PIV1, 2) Yes (PIV3)			>4-fold rise serum Ab titer (EIA); OP PCR positive
Rabies virus	Yes	>1 positive test: CSF PCR positive; saliva PCR positive		IgG detected in convalescent serum
Rubella virus	Yes	CSF Ab positive		>4-fold rise serum Ab titer if no recent vaccination
Varicella-zoster virus (human herpesvirus 3)	Yes	CSF PCR positive		
WNV	Yes	IgM in CSF; IgM in serum with a WNV PRNT titer >1:20 and >1:4-fold higher than JEV and DENV titer	IgM in serum with a WNV PRNT title >1:20 but <1:4-fold higher than JEV and DENV titer	

*Ab, antibody; CHIKV, chikungunya virus; CSF, cerebrospinal fluid; EIA, enzyme immunoassay; IFA, immunofluorescence assay; OP, oropharyngeal swab sample; PRNT_90_, 90% plaque reduction neutralization test; HAdV, human adenoviruses; DENV, dengue virus; JEV, Japanese encephalitis virus; PIV, parainfluenza virus; WNV, West Nile virus. Blank cells indicate not applicable.

### Etiologic Classification

Definitions were created to define the etiologic link between identified pathogens and encephalitis; the etiology for each case was classified as confirmed, probable, possible, or unknown (Table 1). Each case could be assigned >1 etiology. The classification system considered the nature of an agent (well-established cause of encephalitis or not) and etiologic significance of a given positive laboratory test result.

Etiology was considered confirmed for cases with positive results for PCR, culture, antigen, or pathogen-specific IgM in CSF for a pathogen considered to be a well-established cause of encephalitis. For arboviruses commonly associated with encephalitis (i.e., JEV or West Nile virus), documentation of acute infection in paired serum samples was considered the diagnostic standard and therefore was considered confirmatory. 

Etiology was considered probable for cases with positive results that were strongly suggestive but not considered confirmatory and not clearly established as diagnostic for encephalitis in all situations or cases for which a pathogen not generally established as a cause of encephalitis was detected in CSF. For example, etiology would be considered probable if PCR of CSF was positive for lymphotropic agents (e.g., cytomegalovirus, Epstein-Barr virus [EBV]) or if serum testing results provided evidence of acute infection for pathogens known to be associated with encephalitis, such as *Orientia tsutsugamushi*, *Bartonella henselae*, and *Mycoplasma pneumoniae*. 

Etiology was considered possible for cases with no laboratory evidence of CNS involvement but some evidence of acute infection that suggested a potential etiologic role in encephalitis, such as a >4-fold rise in antibody titer for enteroviruses or an oropharyngeal swab sample positive for influenza or parainfluenza viruses. In addition, etiology was considered possible for cases for which information to determine etiology of encephalitis (e.g., human adenoviruses 40 and 41) was insufficient, even if detected in CSF (*27*). Etiology was considered unknown for cases for which all testing results were negative.

In an effort to differentiate between cases of encephalitis and meningoencephalitis, we defined a subset of patients who met the case definition and specifically had CSF pleocytosis and stiff neck as having meningoencephalitis. To provide a complete description of all patients who met the case definition, we retained the 8 patients for whom a sole confirmed bacterial agent commonly associated with bacterial meningitis was found. Pertinent analyses were performed with and without these 8 patients. For 1 patient for whom a bacterial etiology was confirmed, a viral etiology (JEV) was also confirmed; thus, this patient was not excluded from either analysis.

### Statistical Analyses

We present descriptive data with case counts and frequencies. We used the Wilcoxon rank-sum test to compare continuous variables between the confirmed/probable and possible/unknown etiologic groups. The prevalence of categorical variables was compared by using χ^2^ analysis or the Fisher exact test. We considered 2-sided p values <0.05 to be statistically significant. Statistical analyses were performed by using SPSS Statistics for Windows version 21.0 (IBM Corp., Armonk, NY, USA).

## Results

**Figure 1:**
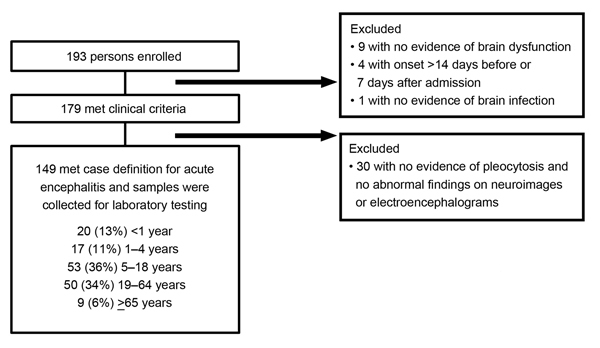
Schematic of enrolled patients who met case definition for inclusion in study of patients with encephalitis, Thailand, 2003–2005.

**Figure 2:**
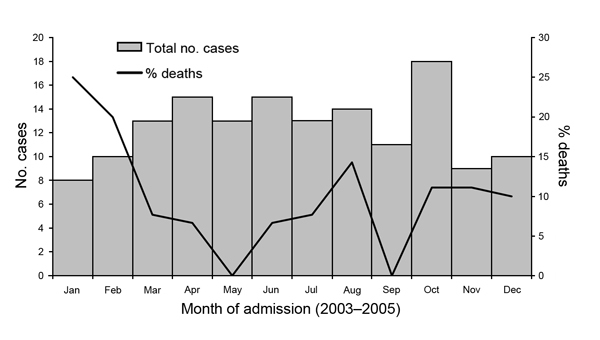
Month of admission for 149 patients with encephalitis, Thailand, 2003–2005.

We enrolled 193 patients, among whom 149 (77%) met the case definition for acute encephalitis (Figure 1). Of the 149 acute encephalitis patients, CSF pleocytosis was found for 125 (84%), abnormal neuroimages for 80 (54%) (45 brain MRI, 55 brain CT, 6 cranial ultrasonography), and EEG findings consistent with encephalitis (categories not mutually exclusive) for 28 (19%). Of the 149 patients, 84 (56%) were male and median age was 12 (range 0–83) years. A median of 5 patients were admitted each month, varying somewhat by season (Figure 2). A total of 30 (20%) patients were from Songkhla; 73 (49%) reported having lived in a city or town in the past 3 months. Median time between onset of neurologic symptoms and admission was 1 day (range 12 days before admission to 7 days after), and median length of hospital stay was 15 (range 1–180) days. A total of 22 (15%) patients had an underlying condition (e.g., HIV infection, malignancy, diabetes mellitus). Of 3 main antimicrobial treatments given empirically during hospitalization, acyclovir was given to 62 (42%) patients; a third- or fourth-generation cephalosporin to 97 (65%), 16 of whom also received a carbapenem; doxycycline to 10 (7%); at least 1 of these drugs to 122 (82%); and all 3 drugs to 4 (3%). A total of 15 (10%) patients died.

With regard to hospital testing, blood culture was performed for 129 (87%), CSF culture for 141 (95%), and both cultures for 123 (83%). Growth occurred on 21 (16%) blood cultures and 19 (13%) CSF cultures (Table 2); pathogens grew on both cultures for 7 patients. No patients had positive malaria testing results, and 8 (5.4%) had positive HIV results.

**Table 2:** Clinical testing results for 149 patients meeting the case definition of encephalitis, Thailand, 2003–2005*

Hospital test	No. positive/ no. tested (%)	Organisms identified (no. if >1)
Blood		
Culture	21/129 (16)	*Acinetobacter* spp., *Escherichia coli,*^1^ gram-negative bacilli,† gram-positive cocci (2),^2^† *Haemophilus influenzae*,^3^† *Klebsiella pneumoniae, Proteus mirabilis/Staphylococcus *coagulase negative,* Pseudomonas aeruginosa *(2),^4^ *Salmonella* group D,^5^† *Staphylococcus aureus *(3),^6^ *Staphylococcus* coagulase negative (5), *Streptococcus pneumoniae,*^7^* Streptococcus *not group A, B, D
Malaria smear	0/123	
VDRL	1/124 (1)	
HIV‡	8/137 (6)	
Toxoplasmosis antibody	0/2	
Cerebrospinal fluid		
Culture	19/141 (13)	*Bacillus* spp.,^6^* Cryptococcus, Cryptococcus/Acinetobacter* spp. (2), *E. coli*,^1^ gram-negative nonfermenting bacilli† gram-positive cocci in clusters,† *Haemophilus influenzae *(2),^3^† *Neisseria meningitidis, P. *a*eruginosa*,^4^* Salmonella* group D,^5^* S. aureus*, *Staphylococcus*, coagulase negative (3), *S. pneumoniae *(2),*^2,7^* *Streptococcus viridans*
India ink stain	2/59 (3)	
Cryptococcal antigen test	1/31 (3)	
VDRL	0/6	
Latex agglutination		
Group B *Streptococcus*	1/43 (2)	
* Neisseria meningitidis*	0/54	
* S. pneumoniae*	3/55 (5)	
* H. influenzae*	3/55 (5)	
* Escherichia coli*	0/48	
PCR		
Herpes simplex virus	1/38 (3)	
Varicella-zoster virus	1/5 (20)	
* Mycobacterium tuberculosis*	4/30 (13)	
Sputum smear or culture		
* M. tuberculosis*	1/21 (5)	

*Superscript numbers indicate patients positive on blood and CSF culture for specified pathogens. GPA, gelatin particle agglutination; VDRL, Venereal Disease Research Laboratory. Blank cells indicate organism not applicable. †Further identification not done. ‡6 were positive by ELISA (3 also by GPA, 2 also by GPA and immunochromatography, and 1 also by immunochromatography), 1 by GPA only, and 1 by an unrecorded assay.

For most patients, specimens were available for additional testing at the reference laboratories (Thailand National Institute of Health and CDC). CSF specimens were available for 147 (99%) (median 3.1, range 0.8–13 mL), acute-phase serum for 145 (97%) and convalescent-phase serum for 129 (87%) (median time between sample collection 24 [range 14–39] days), oropharyngeal swab samples for 141 (95%), saliva for 138 (93%), feces for 119 (80%), and urine for 143 (96%) patients.

### Case Designation by Etiologic Classification

Of the 149 cases, etiology was confirmed for 37 (25%), probable for 17 (11%), possible for 44 (30%), and unknown for 51 (34%) (Table 3). Among confirmed etiologies, JEV was identified in 15 patients and dengue virus in 2. In >1 patient, infection with the following was also identified: enteroviruses (n = 6), *Cryptococcus* spp. (n = 3), *Haemophilus influenzae* (n = 3), *Streptococcus pneumoniae* (n = 3), and varicella-zoster virus (VZV; n = 2). Among case-patients whose illness met the definition for confirmed etiology, a bacterial pathogen commonly associated with meningitis was confirmed for 8 patients (*H. influenzae* for 3, *S. pneumoniae* for 3, *Neisseria meningitidi*s for 1, and *Escherichia coli* for 1). 

**Table 3:** Final classification of cases of encephalitis into etiologic category Thailand, 2003–2005*

Pathogen	Etiologic classification category			Total
Confirmed	Probable	Possible
Adenovirus	0	0	7†	7
*Bartonella henselae*	0	0	0	0
Chikungunya virus	0	0	0	0
*Cryptococcus* spp.	3	0	0	3
Dengue virus	2	0	0	2
Epstein-Barr virus	0	3	0	3
Enteroviruses	6‡	0	24§	30
*Escherichia coli*	1	0	0	1
Herpes simplex virus 1/2	1	0	0	1
Human herpesvirus 6	0	0	0	0
Human herpesvirus 7	0	0	1	1
*Haemophilus influenzae*¶	3	0	0	3
HIV	0	0	8	8
Influenza viruses	0	1	5	6
Japanese encephalitis virus#	15	6	0	21
Malaria	0	0	0	0
Measles virus	0	0	10	10
Mumps virus	0	0	8	8
*Mycoplasma pneumoniae*	0	3	1	4
Nipah virus	0	0	0	0
*Neisseria meningitidis*	1	0	0	1
Other bacteria	0	0	21**	21
*Orientia tsutsugamushi* (scrub typhus)	0	6	0	6
Parainfluenza viruses 1,2,3	0	0	5	5
Parasites, other	0	0	0	0
Rabies virus	0	0	1††	1
*Rickettsia conorii* (spotted fever)	0	3	0	3
Rubella	0	0	4	4
*Salmonella* group D	1	0	0	1
*Streptococcus pneumoniae*	3	0	0	3
*Treponema pallidum*	0	1‡‡	0	1
Varicella-zoster virus	2	0	0	2
West Nile virus	0	0	0	0
Total no. pathogens detected	38	23	95	156
Total no. patients	37	17	44	98

*Strategy used is same as that shown in Table 1. Pathogen categories are not mutually exclusive. †Only 1 in a cerebrospinal fluid sample was positive by PCR and it was adenovirus type 40. ‡Echovirus 9 (n = 3); echovirus 27 (n = 2); echovirus 30 (1). §Enterovirus 71 (n = 3); enterovirus, untyped (n = 21). *¶*Not typed. #This number is 1 less confirmed Japanese encephalitis case than previously published (*29*). The excluded patient had onset of neurologic symptoms 18 d after hospital admission. **See Table 2 for organisms, excludes coagulase-negative *Staphylococcus.* ††Record says never vaccinated. ‡‡Serum Venereal Disease Research Laboratory testing only.

Among patients for whom etiology was probable, *O. tsutsugamushi*, which causes scrub typhus, was found in 6 patients (5 became ill while in central Thailand and were hospitalized in Bangkok); JEV in 6, EBV in 3 (concurrent with *Cryptococcus* spp. in 1 and VZV in 1); *M. pneumoniae* in 3; and *Rickettsia conorii*, which causes spotted fever, in 3 patients. Among the 54 patients for whom etiology was confirmed/probable, a potentially treatable pathogen was identified for 25 (46%) (*C. neoformans*, *E. coli,* herpes simplex virus, *H. influenzae*, influenza viruses, *M. pneumoniae*, *N. meningitidis*, *O. tsutsugamushi*, *R. conorii*, *S. pneumoniae*, and *Treponema pallidum*). We did not include the patient with confirmed JEV and *Salmonella* group D infection in the treatable category.

Among the 98 patients for whom at least 1 pathogen was identified, a total of 156 pathogens were detected (Table 3); of 40 (41%) patients for whom >1 pathogen was detected, etiology was classified as possible for most. Of the 54 patients for whom etiology was confirmed/probable, >1 pathogen was classified as confirmed/probable for 6 (11%): (JEV/*O. tsutsugamushi*/*R. conorii*, JEV/*R. conorii*, JEV/*Salmonella*, *Cryptococcus* spp./EBV, VZV/EBV, VZV/influenza virus), whereas at least 1 possible etiology was also detected for 28 (52%) (p<0.01). Of 44 patients for whom a possible etiology was identified, >1 possible pathogen was detected for 12 (27%).

Of the 149 patients, 68 (46%) met the definition for meningoencephalitis; for 38 (56%) of these patients, EEG or neuroimaging findings were abnormal, consistent with encephalitis. Of these 38 patients, pathogens considered confirmed/probable etiologic agents were detected for 12 (32%): JEV for 8 (also *R. conorii* for 2 and *O. tsutsugamushi* for 1), *Cryptococcus* for 2, *M. pneumoniae* for 1, and *R. conorii* for 1. Of the 30 remaining patients, pathogens that conferred a confirmed/probable etiologic classification were detected for 16 (53%): JEV for 6, *O. tsutsugamushi* for 4, enterovirus for 3, *N. meningitidis* for 1, herpes simplex virus 1/2 (not distinguished) for 1, and VZV/EBV (positive for both) for 1.

At admission, very few demographic and clinical differences between those with confirmed/probable and possible/unknown etiologies were found (Table 4). Among patients for whom etiology was confirmed/probable, median temperature was higher, loss of consciousness was less common, and stiff neck was more common. When the 8 patients with bacterial meningitis were excluded from analysis, patients for whom etiology was confirmed/probable had a higher median temperature than those for whom etiology was possible/unknown (37.9 vs. 37.7, p = 0.04) and were more likely to have a stiff neck (54% vs. 31%, p = 0.01). Among the 54 patients for whom etiology was confirmed or probable, no differences were found for demographic characteristics and only 1 difference was found for clinical characteristics at admission (Table 4) between those for whom etiology was or was not treatable; temperature was lower among those for whom etiology was treatable (37.9 vs. 39, p<0.01).

**Table 4:** Admission characteristics of 149 case patients with encephalitis, according to etiologic classification, Thailand, 2003–2005*

Characteristic	Etiologic classification		p value
	Confirmed/probable, n = 54	Possible/unknown, n = 95	
Male sex, no. (%)	33 (61)	51 (54)	0.38
Age, y, median (range)	9 (0–74)	17 (0–82)	0.10
Time between onset of neurologic symptoms and admission, d, median (range)	1 (8–4)†	1 (12–7)†	0.35
Time between onset of fever and admission, d, median (range)	4 (31–1)†	4 (35–2)†	0.33
Temperature, °C, median (range)	38.0 (36.0–42.0)	37.7 (35.5–40.2)	0.03
Symptoms during illness, no. (%)			
Respiratory	12 (22)	27 (28)	0.41
Gastrointestinal	23 (43)	36 (38)	0.57
Rash	4 (7.4)	14 (15)	0.30
Glasgow Coma Scale score, no. (%)			0.44
<8	7 (13)	18 (19)	
9–12	13 (24)	27 (28)	
>13	34 (63)	50 (53)	
Mean (SD)	12 (3.2)	12 (3.7)	0.37
Neurologic signs/symptoms, no. (%)			
Alteration of consciousness	35 (65)	63 (66)	0.85
Lethargy	27 (50)	45 (47)	0.76
Seizure	19 (35)	48 (50)	0.07
Focal neurologic signs	19 (35)	32 (34)	0.89
Personality change	22 (41)	39 (41)	0.97
Somnolence	23 (43)	34 (36)	0.44
Loss of consciousness	8 (15)	28 (30)	0.04
Extreme irritability	9 (17)	23 (24)	0.28
Coma	10 (19)	15 (16)	0.67
Ataxia	2 (3.7)	6 (6.3)	0.71
Headache	29 (54)	45 (47)	0.46
Stiff neck	29 (54)	29 (31)	0.01
Pentobarbital or paralytic medications for intractable seizures	15 (28)	22 (23)	0.53
CSF pleocytosis, no. (%)	49 (91)	76 (80)	0.09
Abnormal MRI/CT/cranial U/S findings, no. (%)	23/31 (74)	57/69 (83)	0.33
Abnormal EEG, no. (%)	6/9 (67)	22/30 (73)	0.69
Length of hospital stay, median days (range)	13 (1–180)	16 (1–128)	0.71
Intensive care unit admission, no. (%)	15 (28)	33 (35)	0.38
Died, no. (%)‡	2 (3.7)	13 (14)	0.09

*CSF, cerebrospinal fluid; EEG, electroencephalogram, MRI, magnetic resonance imaging; CT, computed tomography; U/S, ultrasonography. †Ranges indicate no. days before and after hospital admission. ‡All but 1 patient died while hospitalized.

### Outcomes

A total of 15 (10%) patients died. The mortality rate was lower, but not significantly, among patients for whom etiology was confirmed/probable than for those for whom etiology was possible/unknown (3.9% vs. 14%, p = 0.09; Table 4). When the 8 patients for whom only a confirmed bacterial etiology was found were excluded from analysis, this difference was similar (4.3% vs. 14%, p = 0.14). Among those who died, etiology was confirmed for 2 (*Cryptococcus* spp. in a 5-year-old boy and VZV in a 51-year-old HIV-infected woman). Among those who died, 9 (60%) were male and the median age was 34 years of age (1 was <1 year, 1 was 1–4 years, 4 were 5–18 years, 8 were 19–64 years, and 1 was ≥65 years). The median interval between hospital admission and death (or discharge for survivors) was 6 days (vs. 16 days to discharge, p = 0.3), between onset of neurologic symptoms and death was 10 days (vs. 18.5 days to discharge, p = 0.3), and between fever onset and death was 10 days (vs. 20 to discharge, p = 0.2). Among the 134 patients who survived, 43 (32%) remained hospitalized at the time of the convalescent-phase interview, 86 (64%) had returned home, and 5 (3.7%) were lost to follow-up; 5 patients had a seizure after discharge. Of the 86 persons who returned home, 52 (60%) reported complete cognitive recovery; of these, 45 (87%) of these functioned independently or at the same level of care as before hospitalization.

## Discussion

In Thailand, a wide range of pathogens cause acute encephalitis. In this study, by using a comprehensive approach and advanced diagnostic methods, we identified a confirmed/probable etiology for only 36% of 149 patients, and >1 confirmed/probable pathogen was detected for 11% of these patients. Detection of possible pathogens was so common as to make interpretation challenging. Ten percent of patients died; highest mortality rate was among patients for whom etiology was classified as possible/unknown.

In our study, the most frequently identified pathogen (39% of all confirmed/probable etiologies) was JEV, which is endemic to Thailand; routine infant vaccination was introduced in 2001 (*28*). JEV data from our study have been published (*29*). Among patients for whom etiology was confirmed/probable, the etiologic pathogen was potentially treatable for 48%. Most of these 12 treatable pathogens, except herpes simplex virus, influenza viruses, *M. pneumoniae*, *O. tsutsugamushi*, and *R. conorii*, can be diagnosed in the study hospital laboratories in Thailand through routine CSF and blood culture, Venereal Disease Research Laboratory testing, or rapid antigen tests of CSF. The clinical features did not enable differentiation of specific etiologic agents. For many patients, a standard empiric treatment regimen consisting of a third- or fourth-generation cephalosporin, acyclovir, and doxycycline might be appropriate. The national reference laboratory in Thailand is able to test for all confirmed/probable etiologic agents. 

This study highlights the clinical overlap between encephalitis and meningoencephalitis. Almost half of the patients met the definition for meningoencephalitis, yet for half of those patients, abnormalities detected by EEG or neuroimaging were consistent with encephalitis, and their illnesses were associated with a range of pathogens, some not typically associated with meningitis. Furthermore, we found that patients whose meningitis was caused by common bacteria, such as *S. pneumoniae* or *H. influenzae*, sometimes met the case definition but did not meet the criteria for meningoencephalitis (pleocytosis and stiff neck). We reported laboratory results for all patients who met the encephalitis case definition, and we also repeated the analyses excluding the 8 patients with only a confirmed etiology of bacteria commonly associated with meningitis, which minimally affected the results. It could be argued that including pleocytosis in the case definition may have resulted in a predilection for enrolling patients with meningoencephalitis and/or meningitis; however, pleocytosis has commonly been included in the case definition for studies of encephalitis (*24*), and including it was necessary for our study because we were uncertain whether neuroimaging would be routinely performed.

Several pathogens that cause encephalitis were notably absent, including chikungunya, Nipah, and West Nile viruses; *B. henselae*; and malaria parasites. *B. henselae* infection occurs in Thailand, although it has not been widely studied (*30*). Because of extensive vector control efforts in Thailand, malaria parasite transmission is limited to areas along Thailand’s borders with Burma and Cambodia, so it was not surprising that no cases of malaria were identified in Bangkok and Songkhla (*31*). Nipah virus has caused outbreaks of encephalitis in humans in Malaysia and Bangladesh; although it has been found in bats in Thailand, it has not yet been identified in humans (*32*,*33*). West Nile virus has also not yet been identified in Thailand.

Enteroviruses were found in CSF of 6 children 3 months to 10 years of age. Enteroviruses commonly cause aseptic meningitis and have also been clearly demonstrated to cause encephalitis. In Asia, epidemics of enterovirus 71 infection causing severe central nervous disease have occurred (*34*); however, enterovirus 71 was not detected in the CSF of any patient in our study. Consistent with reports in the literature, we found 2 cases of dengue virus infection (*35*). We found 6 cases of infection with *O. tsutsugamushi,* the etiologic agent of scrub typhus, which is prevalent in Southeast Asia although not thought to be common in Bangkok. Thus, the fact that 5 patients became ill while in central Thailand suggests that physicians should consider this pathogen in areas outside the known disease-endemic southern provinces. Most cases of scrub typhus, and spotted fever caused by *R. conorii* (which we identified in 3 patients), can be treated effectively with doxycycline (*36*). Unfortunately, most hospital laboratories do not routinely perform diagnostic serologic testing for these infections, so they might often not be suspected unless a prominent eschar or rash is visible. EBV was found in the CSF of 3 patients, concomitant with another pathogen for 2 patients. In these cases, EBV detection probably represented reactivation of latent EBV in the setting of a primary central nervous system infectious agent (*37*). Three cases of encephalitis were associated with *M. pneumoniae*, all in children 6–14 years of age, consistent with other study findings (*38*,*39*).

Comprehensive etiologic studies present many challenges and have limitations. In this study, CSF diagnostics were the most compelling approach for identifying an etiology; yet in some patients, CSF or other specimens were inadequate for complete testing for all agents. We used consensus primers to detect herpesviruses by PCR, but this method is probably less sensitive than one that uses specific primers for each herpesvirus. Feces and oropharyngeal swabs samples were not tested for enteroviruses, but these samples can be more likely to yield this pathogen. Last, we did not investigate noninfectious forms of encephalitis, such as anti–*N-*methyl-D-aspartate receptor and voltage-gated potassium channel antibody encephalitis, which have been shown in recent studies to cause encephalitis with poor outcomes (*7*,*40*).

Encephalitis is a severe disease that can cause substantial illness and death. Despite the high proportion of patients for whom large volumes of CSF and acute- and late convalescent–phase serum were tested and a broad range of routine and sophisticated diagnostic testing was performed, the etiology for one-third of the patients remained unknown and for another one-third was classified only as possible. The proportion of cases for which etiology was unknown in this study was slightly lower than that in other studies, perhaps because so many diagnostic tests were used (*1*,*10*–*12*,*26*). Because laboratory testing was performed simultaneously rather than sequentially, testing bias was reduced; however, because of multiple positive results, the complexity of interpretation was increased.

Although Thailand is a middle-income country with trained neurologists and more sophisticated medical and laboratory tools for diagnosing encephalitis than are found in many other countries in Southeast Asia, it still lacks the ability to routinely identify major causes of encephalitis. This study highlights the continuing role of Japanese encephalitis and other potentially preventable or treatable diseases in Thailand, such as those caused by the rickettsiae *O. tsutsugamushi* and *R. conorii*. Despite intensive efforts to diagnose cases, this study also emphasizes the need for improved diagnostic strategies and geographically appropriate clinical testing algorithms for adults and children with encephalitis in Southeast Asia. Such strategies and algorithms were recently presented by the International Encephalitis Consortium (*24*) to provide a standardized approach for the evaluation of patients suspected of having encephalitis. Such an approach will facilitate worldwide research collaboration and enable clinicians to provide appropriate clinical care for patients with this severe and often devastating neurologic syndrome.

Technical Appendix Specimen testing algorithm for all 149 cases of encephalitis, Thailand, 2003–2005.
