# Supplementary material for: Infectious Causes of Encephalitis and Meningoencephalitis in Thailand, 2003–2005
Source: Emerg Infect Dis. 2015 Feb;21(2):280–9. doi: 10.3201/eid2102.140291 (PMC4313633; doi:10.3201/eid2102.140291)
Supplement: Technical Appendix — Specimen testing algorithm for all 149 cases of encephalitis, Thailand, 2003–2005. [file 14-0291-Techapp-s1.pdf]

Article DOI: http://dx.doi.org/10.3201/eid2102.140291

Infectious Causes of Encephalitis and Meningoencephalitis in Thailand, 2003–2005

Technical Appendix

Appendix Table. Specimen testing algorithm for all 149 cases of encephalitis, Thailand, 2003–2005

Specimen type (total volume)	Volume of aliquot	Laboratory	Testing in order of priority[a]
CSF (6.5ml)	1.5 ml	Hospital	Cell count<br>Protein/glucose<br>Other (Gram stain, bacterial culture, mycobacterial culture, bacterial latex agglutination, Cryptococcal antigen test, India ink stain, VDRL assay, microscopy)<br>PCR for herpes simplex virus, varicella zoster virus and *Mycobacterium tuberculosis*
	1.5 ml	Thai NIH	Japanese encephalitis and dengue viruses (IgM)<br>Enteroviruses (isolation in culture)<br>Herpes simplex viruses 1 and 2 (IgM)<br>Measles, mumps, rubella viruses (CSF Ab)<br>Rabies virus (PCR)
	1.8 ml	CDC	16S ribosomal RNA PCR<br>Enteroviruses (PCR)<br>Dengue virus (PCR, IgM)<br>Japanese encephalitis virus (IgM)<br>West Nile virus (IgM)<br>Herpesviruses (PCR)<br>*Mycoplasma pneumoniae* (PCR)<br>Nipah virus (PCR)<br>Pathogen discovery PCR panel[b]
	0.5 ml	CDC	Store for confirmatory testing
	1.2+ ml	Thai NIH	Storage in 1 ml aliquots
Blood	2.5 ml child<br>5.5 ml adult	Hospital	Malaria thick and thin smears<br>VDRL, specific treponemal Ab<br>HIV serology<br>Bacterial culture
Serum (10ml)[c]	1.5 ml	Thai NIH	Toxoplasmosis serology<br>Enteroviruses (serology)<br>Herpes simplex viruses 1 and 2, VZV (serology)<br>Japanese encephalitis and dengue viruses (serology)<br>Measles, mumps, rubella viruses (serology)<br>Scrub and murine typhus (serology)
	1.8 ml	CDC	Dengue, Japanese encephalitis, Chikungunya, West Nile viruses (IgM, PRNT)<br>Nipah virus (serology)
	0.7 ml	CDC	Adenoviruses (serology)<br>*Bartonella henselae* (serology)<br>Influenza viruses (serology)<br>Measles, mumps rubella viruses (serology)<br>*Mycoplasma pneumoniae* (serology)<br>Parainfluenza viruses 1, 2 and 3 (serology)<br>Rabies virus (serology)

Specimen type (total volume)	Volume of aliquot	Laboratory	Testing in order of priority[a]
			Rickettsia spp. (serology)
	1.0 ml	CDC	Store for confirmatory testing
	5.0 ml	Thai NIH	Storage in 1 ml aliquots
Saliva	0.7 ml	CDC	Rabies virus (PCR)
Oropharyngeal swab	One swab in viral transport media	CDC	Adenovirus (PCR)
			Influenza viruses (PCR)
			Mycoplasma pneumoniae (PCR)
			Parainfluenza viruses 1, 2, 3 (PCR)
Urine	10 ml	Thai NIH	Store for future testing
Stool	10-20 g	Thai NIH	Parasites (microscopy)
Sputum		Hospital	*Mycobacterium tuberculosis*

[a] In hospital laboratories, specialized testing for certain organisms may have been performed only if suspected clinically; in Thai NIH and CDC reference laboratories, the testing algorithm was prioritized as shown according to available specimen volume.
[b]Consensus PCR primers for Alphaviruses, Flaviviruses, Bunyaviruses, Herpesviruses group A, Herpesviruses group B, Adenoviruses, VZV
[c]12.5-22.5 ml whole blood from children <5 years and 25.5 ml for all other ages.
Abbreviations: VDRL=venereal disease research laboratory; Ab=antibody; PCR=polymerase chain reaction; PRNT= plaque reduction neutralization test; VZV, varicella zoster virus.
